# Physical Activity among Adults Residing in 11 Countries during the COVID-19 Pandemic Lockdown

**DOI:** 10.3390/ijerph18137056

**Published:** 2021-07-01

**Authors:** Kele Ding, Jingzhen Yang, Ming-Kai Chin, Lindsay Sullivan, J. Larry Durstine, Verónica Violant-Holz, Giyasettin Demirhan, Nara R.C. Oliveira, Biljana Popeska, Garry Kuan, Waheeda Khan, Jianhui Dai, Xia Xu, Zornitza Mladenova, Govindasamy Balasekaran, Gary A. Smith, on behalf of Global Community Health-COVID-19 Collaborative Research Team

**Affiliations:** 1 School of Health Science, College of Education Health & Human Service, Kent State University, Kent, OH 44242, USA; kding@kent.edu; 2 Center for Injury Research and Policy, The Abigail Wexner Research Institute at Nationwide Children’s Hospital, Columbus, OH 43205, USA; Gary.Smith@nationwidechildrens.org; 3 Foundation for Global Community Health, Las Vegas, NV 89012, USA; 4 Discipline of Children’s Studies, School of Education, National University of Ireland, H91 Galway, Ireland; lindsaymarie.sullivan@nuigalway.ie; 5 Department of Exercise Science, Norman J. Arnold School of Public Health, University of South Carolina, Columbia, SC 29208, USA; ldurstin@mailbox.sc.edu; 6 Department of Didactics and Educative Organization, University of Barcelona, 08015 Barcelona, Spain; vviolant@ub.edu; 7 Department of Physical Education and Sport Teaching, Faculty of Sport Sciences, Hacettepe University, Ankara 06800, Turkey; demirhan@hacettepe.edu.tr; 8 Department of Human Movement Sciences, Federal University of Sao Paulo, Santos 11015, Brazil; nara.rejane@unifesp.br; 9 Faculty of Educational Sciences, Goce Delcev University, 2000 Stip, North Macedonia; biljana.popeska@ugd.edu.mk; 10 Exercise and Sports Science, School of Health Sciences, Universiti Sains Malaysia, Kubang Kerian 16150, Malaysia; garry@usm.my; 11 Faculty of Behavioural Sciences, SGT University, Gurugram 122505, India; profwkhan@gmail.com; 12 School of Physical Education and Sports, Soochow University, Suzhou 215021, China; sddjh@suda.edu.cn; 13 Hubei Key Laboratory of Sport Training and Monitoring, Wuhan Sports University, Wuhan 430079, China; xuxia@whsu.edu.cn; 14 Association of Touristic Animators, 1000 Sofia, Bulgaria; z.mladenovaz@gmail.com; 15 National Institute of Education, Nanyang Technological University, Singapore 637616, Singapore; govindasamy.b@nie.edu.sg

**Keywords:** changes in physical activity, governmental policy responses, insufficiently active

## Abstract

During the novel coronavirus (COVID-19) pandemic, physical activity (PA) behaviors were altered worldwide due to public health measures such as “lockdown.” This study described PA among adults residing in 11 countries during COVID-19 lockdown and examined factors associated with PA engagement. We conducted a cross-sectional anonymous survey among adults (≥18 years old) in 11 countries (Brazil, Bulgaria, China, India, Ireland, Malaysia, North Macedonia, Singapore, Spain, Turkey, United States). Of 11,775 participants, 63.7% were female and 52.8% were 18–34 years old. More than 40% of participants were insufficiently active (43.9%) and reported a decrease in their PA during lockdown (44.8%). Statistically significant differences were observed in (1) proportions of participants being insufficiently active, (2) level of PA, and (3) decrease in PA across the 11 countries. More stringent governmental policy responses were associated with greater likelihood of being insufficiently active during lockdown (adjusted odds ratio = 1.22, 95% confidence interval = 1.03, 1.45). Higher depression or anxiety scores were associated with greater likelihood of decreased level of PA during lockdown.We found substantial reductions in PA levels during COVID-19 lockdown across countries. Country-specific PA promotion interventions are needed during this and similar global emergencies.

## 1. Introduction

The novel coronavirus (COVID-19) outbreak resulted in governments worldwide imposing “lockdown,” “stay-at-home,” or “social distancing” policies and restrictions for a prolonged period [1,2]. While these public health measures have slowed the spread of COVID-19 infections, these measures have confined people to their homes and forced them to adapt to a “new normal” way of life [2,3]. This “new normal” had a significant negative impact on the physical and mental health of individuals around the globe, largely due to the uncertainty of exposure to COVID-19 as well as stress and loneliness from social isolation [4,5,6,7,8,9]. Under such circumstances, engaging in daily physical activity (PA) becomes more important than ever as PA is known to help maintain good physical and mental health [10,11,12]. However, lockdown and stay-at-home policies, coupled with the closure of recreation facilities, city parks, and playgrounds, have made it challenging to engage in the recommended PA levels [13,14].

Existing evidence suggests that regular PA is a cornerstone of health promotion and disease prevention as well as beneficial for helping people cope with difficult times and to fend off infections [10,11,12,13,14,15,16]. The World Health Organization (WHO) guidelines on PA recommend that adults 18 to 65 years old participate in at least 150 min of moderate-intensity, or 75 min of vigorous-intensity aerobic PA, or an equivalent combination of both each week [11]. However, with individual daily routines shifting dramatically during the COVID-19 pandemic (i.e., working from home, homeschooling, and avoiding unnecessary trips outside homes), along with the closure of fitness and health centers, gyms, and outdoor recreation facilities, meeting the WHO PA recommendations has become challenging [17,18,19,20,21]. Several prior studies, conducted in single and multiple countries, documented PA levels during the COVID-19 lockdown using self-reported measures or wearable activity trackers; the findings showed significant reductions in PA levels and increased sedentary behavior among adults during the COVID-19 lockdown [18,19,20,21,22,23,24]. Furthermore, although creative solutions for being active at home or in other physically distanced settings during lockdown have been promoted by governments, public health officials, and sport managers [11,13,14], little information is available on how the COVID-19 lockdown and associated restrictions have influenced PA engagement across countries and what strategies individuals residing in different countries have used to remain physically active during the lockdown period.

To address this gap in the literature, the present study described PA undertaken by adults ≥18 years residing in 11 countries during COVID-19 lockdown, and examined the factors associated with levels of PA engagement. Specifically, we investigated the associations of country-level COVID-19 risk (i.e., increase in confirmed cases per million people and governmental policy responses to COVID-19), mental health symptoms (i.e., depression and anxiety symptoms), and demographic factors with PA engagement, including physical inactivity (i.e., insufficiently active), level of PA (low, moderate, or high), and change in PA (decrease, increase, or no change) during the COVID-19 lockdown. Additionally, among participants who reported engaging in PA during lockdown, we examined the location and mode of their PA engagement. The findings of this study contribute to the current literature on the potential impact of the COVID-19 lockdown on PA levels among adults from a global perspective [16,25,26].

## 2. Materials and Methods

### 2.1. Study Design and Participants

We conducted a cross-sectional, multicountry survey study among a convenience sample of adults ≥18 years old who resided in 1 of 11 countries: Brazil, Bulgaria, China, India, Ireland, Malaysia, North Macedonia, Singapore, Spain, Turkey, and the United States (US). We established a collaborative research group that comprised 11 research teams (one team from each country), ranging in size from three to five members, with one designated leader per country. The US team developed the study protocol which was followed and carried out by each team in their respective country. The US team was also responsible for coordinating research activities between countries. To ensure the study procedures were implemented consistently across the 11 countries, we used the same valid and reliable instruments in all participating countries and limited the data collection period to 30 days for each country from start to end date of active study enrollment, and between 1 June to 31 August 2020. More details about the study design and procedures are described elsewhere [27]. The study first received ethical approval from the Institutional Review Board at Nationwide Children’s Hospital (in the US, ID = STUDY00001110). Each of the 10 remaining country teams also received ethical approval from their respective Institutional Review Board. By completing the survey, participants agreed to participate in the study.

### 2.2. Survey Instrument 

The US team developed the initial survey instrument in English, based on existing validated instruments [28,29,30] and published studies on the impact of COVID-19 on PA [6,11,13,17]. The US team then sent the initial instrument, along with written instructions, to each participating team for expert review. The survey instrument underwent an iterative review process from each team and was continually revised until all teams agreed on the draft of the English version of the survey. The instrument in English was then pilot tested in the participating countries (n = 131 valid returned surveys) and modified based on testing results. Following pilot testing, team leaders provided additional feedback prior to finalizing the English version of the survey instrument. The final survey instrument included 73 questions, including skip-pattern questions. The seven countries that used non-English versions of the survey had the final survey translated and back translated by independent bilingual professionals to ensure accuracy before use. In total, we used surveys in eight languages: Bulgarian, Simplified Chinese, English, Macedonian, Malay, Brazilian Portuguese, Spanish, and Turkish [27].

### 2.3. Procedures

Team leaders from each country sent an invitation for this study, including the link to an anonymous online survey (i.e., Qualtrics), to potential participants via email and/or posted the study flyer on their personal and/or institution’s social media accounts (e.g., Facebook, Twitter, WeChat). The first page of the survey included the study eligibility criteria (≥18 years old). After invited participants confirmed their eligibility, they were directed to review additional information about the study before proceeding to the survey. The survey took about 15 min to complete. Following completion of data collection, each team downloaded their country’s survey data and securely sent data to the US team for management and analysis [27]. We received 15,529 surveys from the 11 countries. Of these 15,529 surveys, 1550 (10.0%) surveys were removed because 90% or more of the data points were missing. An additional 2204 (14.2%) surveys were excluded, including 288 participants (1.9%) who rated their general health as either “poor” or “missing,” and 1916 participants (12.3%) who did not respond to any of the three questions asked about their engagement in vigorous PA, moderate PA, or walking during the lockdown period. The final sample for this study included 11,775 surveys.

### 2.4. Study Variables and Measures

*PA* was measured using the seven-item short form of the International Physical Activity Questionnaire (IPAQ). The IPAQ has been validated among individuals 15 to 69 years old with established concurrent and criterion validity, in various languages, and has a test–retest reliability of 0.80 or higher [29,30,31,32]. Participants were asked to report the leisure time PA (e.g., recreation, exercise, or sport) that they had engaged in during lockdown. Specifically, participants were asked to report the frequency (measured in days per week) and duration (time per day) for each of the three specific intensity levels: (1) vigorous-intensity activities, (2) moderate-intensity activities, and (3) walking [29]. Participants were instructed not to include activities related to their job, transportation, or housework. To estimate total weekly metabolic equivalent of task (MET)-minutes, we multiplied the total minutes of vigorous-intensity PA, moderate-intensity PA, and walking per week with established corresponding average MET coefficients in IPAQ (8, 4, and 3.3, respectively) [30,31]. We then classified the weekly PA level into four categories using the combination of total days, minutes, and MET-minutes of PA per week based on the official IPAQ short form guidelines: (1) none, (2) low-, (3) moderate-, or (4) high-level of PA [33]. We further classified participants who were in the none or low-level of PA category as insufficiently active.

*Change in PA during lockdown* was assessed by asking participants to report changes in their level of PA engagement during lockdown as compared to their level of PA before the COVID-19 pandemic, with three response categories: increase, decrease, or no change. We used a dichotomous variable (decrease vs. otherwise) in our regression models.

*Location and mode of PA during lockdown* was assessed among participants who reported that they engaged in PA during lockdown, using five questions: (1) the location of their PA (indoor vs. outdoor; home vs. public place), (2) with whom, if anyone, they engaged in PA, (3) frequency of using online programs to assist their exercise, (4) frequency of using PA as a coping strategy, and (5) frequency of encouraging others to engage in PA during lockdown.

*Country-level COVID-19 risk* was measured using two variables based on publicly available data published by Our World in Data [34]. The first variable, *increase in confirmed cases* per million people, was calculated for each participating country as the average change in the cumulative number of confirmed COVID-19 cases per million people in a country between the date the participating country reached its 100th case and August 31, 2020 (the last day of our study data collection) [27]. The second variable, *governmental policy responses* to COVID-19, was determined for each country using the average daily Government Stringency Index (GSI) score between the date the participating country reached its 100th case and 31 August 2020 [27]. GSI is a daily composite score covering nine policy areas (e.g., school closures, workplace closures, and travel bans), with a possible score from 0 to 100 (0 = no policy response to 100 = the strictest policy response) [34]. Since GSI scores were not available for North Macedonia, we used the average scores of its five neighboring countries (Albania, Bulgaria, Greece, Kosovo, and Serbia) instead.

*Anxiety and depression symptoms* were measured using the valid Adult Patient-Reported Outcomes Measurement Information System (PROMIS)^®^ (PROMIS^®^ Health Organization, Evanston, IL, USA) Short Form v1.0–Anxiety 4a (Cronbach’s alpha of 0.93) and PROMIS^®^ Short Form v1.0–Depression 4a (Cronbach’s alpha of 0.95) in English and seven non-English versions, all of which were provided by the PROMIS^®^ Health Organization [28,35,36]. Each scale included four questions on a five-point Likert-scale (range = 4 to 20), with higher scores representing greater anxiety or depression symptoms. We asked participants to respond to the questions based on their feelings and thoughts during their country’s lockdown. For data analysis, we converted the original total raw scores to T-scores [28].

*Demographic variables* included sex, age group, highest level of education completed, marital status, living with at least one person ≥65 years old during lockdown, having at least one child in the household during lockdown, being required to go out of the home to work during lockdown, and general health status before COVID-19.

### 2.5. Statistical Analysis

We examined the distributions of PA engagement, including PA level (low, moderate, or high), being insufficiently active (yes vs. no), and change in PA (decrease, increase, or no change) during lockdown by country using chi-square tests. We also described the location and mode of PA engagement among individuals who reported that they engaged in PA during lockdown. To examine factors associated with the proportions of participants being insufficiently active or reporting a decrease in their PA during lockdown, we used Hierarchical Generalized Linear Models with dichotomous outcomes to estimate the odds of being insufficiently active or odds of reporting a decrease in PA by country-level COVID-19 risk, mental health symptoms, and demographic variables, using SAS PROC GLIMMIX procedure. Our null models tested the outcome of being insufficiently active (or a reported decrease in PA) as a dichotomous variable without fixed factors. Respondents were nested to their respective countries as a random factor to estimate between country variations. We used intralevel correlation coefficients (ICC) for this purpose. We then tested a full model by including country-level COVID-19 risk, mental health symptoms, and demographic variables as fixed factors. Akaike information criterion (AIC) and Bayesian information criterion (BIC) were used to determine the model fit and improvement from the null model to the full model [37]. We conducted data analyses between 1 December 2020, and 10 February 2021, using SAS version 9.4 (SAS Inst., Cary, NC, USA). We set the significance level at α = 0.05.

## 3. Results

### 3.1. Characteristics of Study Participants

Of the 11,775 participants included, 63.7% were female (n = 7498,), 52.8% were 18–34 years old (n = 6100), 62.9% had a bachelor’s degree or higher (n = 7392), and 49.1% were single (n = 5783) (Table 1). China had the greatest number of participants (n = 1952, 16.6%) followed by Brazil (n = 1432, 12.2%) and Turkey (n = 1360, 11.6%). The overall mean scores (standard deviation (SD)) of anxiety and depression symptoms were 56.4 (9.7), and 52.2 (9.7), respectively.

### 3.2. PA during Lockdown

**Figure 1:**
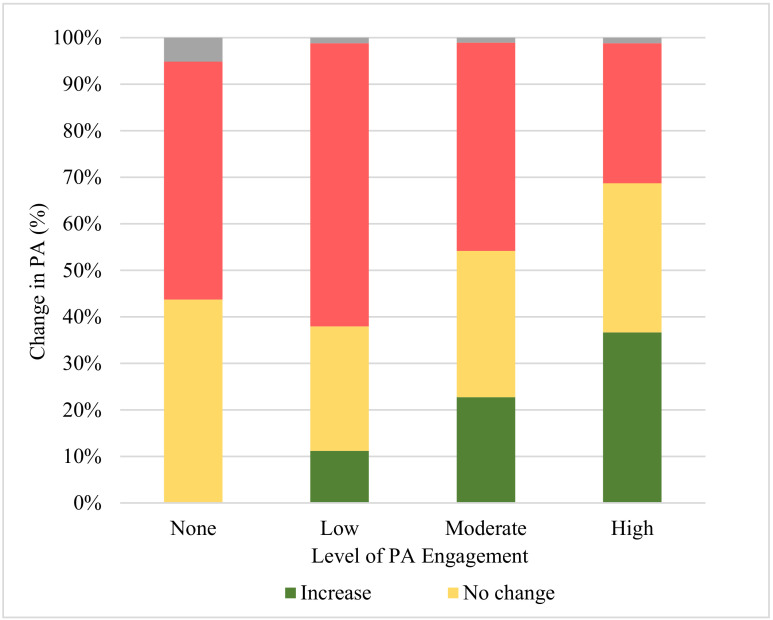
Change in PA by current level of PA engagement (n = 11,775). Note: The denominators of this figure are current level of PA engagement. A chi square test was conducted to test the association between current level of PA engagement and the change in PA, with X^2^ = 1848.6 and *p* < 0.0001.

More than 40% of participants (n = 5169, 43.9%) were insufficiently active during lockdown; this included 23.6% of participants (n = 2780) who reported engagement in no PA and 20.3% of participants (n = 2389) who reported a low level of PA (Table 2). Approximately one-third of participants (n = 3814) engaged in a high level of PA during lockdown, while about one-quarter of participants (n = 2792) engaged in a moderate level of PA. More than 40% of participants (n = 5273, 44.8%) reported a decrease in their PA during lockdown, while about one in five participants (n = 2522, 21.4%) reported an increase in their PA during lockdown. Participants engaging in low PA level during lockdown were more likely to report that their PA decreased from pre-COVID-19 to lockdown, whereas participants who engaged in a high level of PA during lockdown were most likely to report an increase in their PA from pre-COVID-19 to lockdown (*p* < 0.0001) (Figure 1).

### 3.3. PA during Lockdown by Country

Significant differences were observed in (1) proportions of participants who were insufficiently active (*p* < 0.0001), (2) level of PA (*p* < 0.0001), and (3) decreased PA (*p* < 0.0001) across countries (Table 2). More than half of participants in four of the 11 participating countries were insufficiently active during lockdown (Table 2), including participants residing in Brazil (58.2%), Malaysia (55.2%), China (54.4%), and India (51.3%). The highest average GSI scores were observed in three of these four countries, with scores of 82.9 for India, 75.6 for Brazil, and 73.3 for China. The highest and lowest rate of confirmed COVID-19 cases per million population during the study period were also observed in these four countries, with Brazil having the highest rate (106.3 per million people) and China having the lowest rate (0.3 per million people).

Bulgaria had the lowest proportion of participants who were insufficiently active (21.1%) during lockdown, followed by Ireland (25.0%) and North Macedonia (29.8%). Bulgaria also had the lowest average GSI score (51.7), and a relatively low rate of confirmed COVID-19 cases (14.0 per million population) compared to other participating countries.

Forty percent or more of participants in nine countries (excluding Ireland and Singapore) reported a decrease in PA during lockdown, ranging from 39.9% of participants residing in China or the US to 59.1% of participants residing in Turkey. Participants residing in Ireland (36.1%) and Singapore (30.1%) reported the highest proportions of an increased PA level engagement during lockdown.

### 3.4. Location and Mode of PA during Lockdown

**Figure 2:**
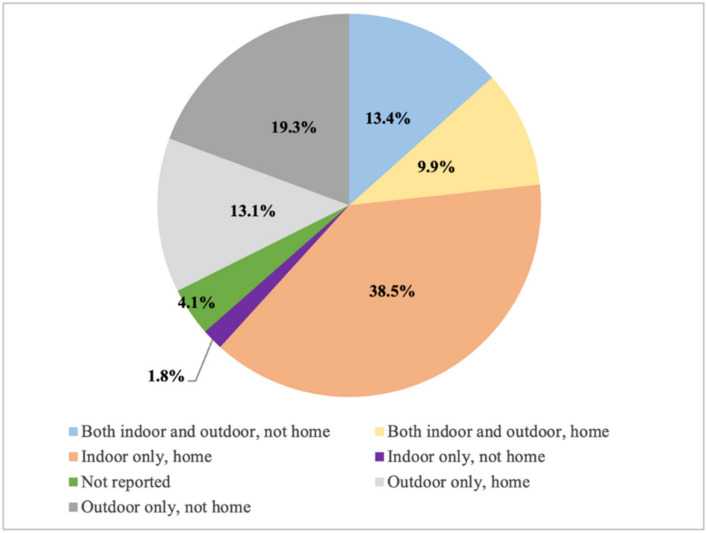
Location of PA among participants who reported engaging in PA during lockdown (n = 8995).

**Figure 3:**
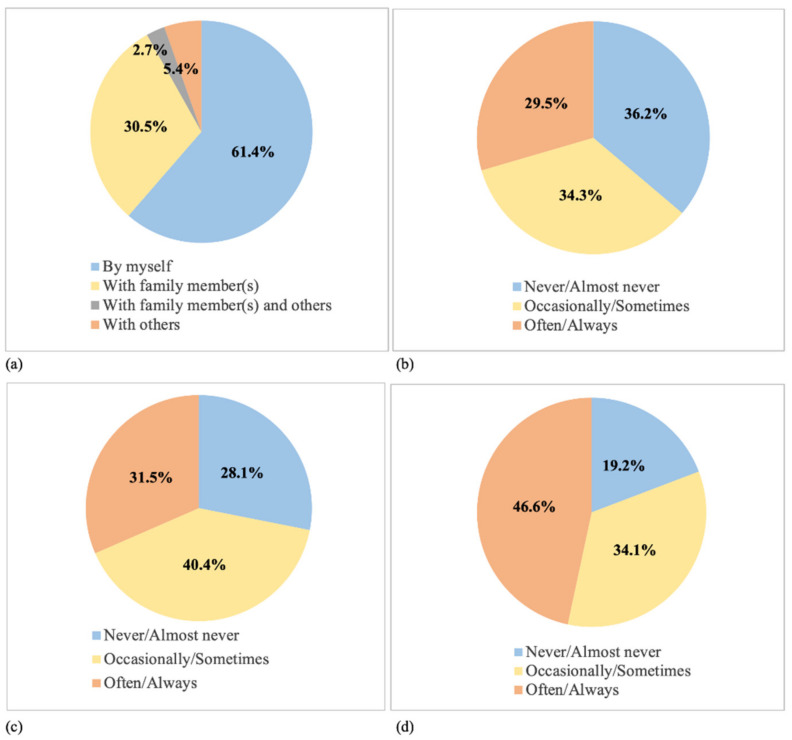
Mode of PA among participants who reported engaging in PA during lockdown (n = 8995): (**a**) with whom PA was engaged, (**b**) frequency of using online programs, (**c**) frequency of using PA as a coping strategy, and (**d**) frequency of encouraging others to engage in PA.

Among the 8995 participants who reported engaging in PA during lockdown, 61.4% (n = 5523) reported engaging in PA at home, with 38.5% (n = 3459) of participants engaging in PA at home indoor only (Figure 2). Of the 8995 participants who engaged in PA during lockdown, 55.7% engaged in outdoor PA either at home or at other public places, 60.5% engaged in PA by themselves, 63.8% used online programs, 46.0% often or always encouraged others to participate in PA, and 71.9% reported that they used PA to cope with COVID-19 (Figure 3). 

### 3.5. Factors Associated with PA Engagement during Lockdown

More stringent governmental policy responses were associated with greater odds of being insufficiently active during lockdown after adjusting for participants’ demographic characteristics and mental health symptoms (Table 3). Every five unit increase in the policy response index score was associated with a 22% increase in the odds of being insufficiently active during lockdown (adjusted odds ratio (AOR) = 1.22, 95% confidence interval (CI) = 1.03, 1.45). However, no associations were observed between country-level COVID-19 risk and a decrease in level of PA engagement during lockdown.

Higher mean depression symptom scores (i.e., every 10-unit increase) were associated with greater odds of being insufficiently active (AOR = 1.44, 95% CI = 1.07, 1.22) and decreased PA level (AOR = 1.15, 95% CI = 1.08, 1.22) during lockdown. Higher mean anxiety scores (i.e., every 10-unit increase) were associated with greater odds of decreased PA level (AOR = 1.18, 95% CI = 1.11, 1.26) during lockdown, but not with being insufficiently active during lockdown.

Participants who were female (AOR = 1.30, 95% CI = 1.19, 1.41), ≥65 years old (AOR = 1.50, 95% CI = 1.18, 1.91), married (AOR = 1.15, 95% CI = 1.02, 1.31), had at least 1 child in the household during lockdown (AOR = 1.17, 95% CI = 1.05, 1.31), or had to go out of the home to work full-time (AOR = 1.16, 95% CI = 1.04, 1.30) had greater odds of being insufficiently active during lockdown compared to their respective counterparts (Table 3). Participants who had a bachelor’s degree or higher education or who reported very good to excellent general health prior to COVID-19 had lower odds of being insufficiently active during lockdown than individuals who had a high school degree or lower education or who reported fair general health prior to COVID-19.

Participants 35–64 years old, or who had a bachelor’s degree or higher education, had greater odds of decreased PA engagement during lockdown than participants 18-24 years old or who had a high school degree or lower education. Participants who had at least one child in the household during lockdown had lower odds of decreased PA during lockdown (Table 3). 

## 4. Discussion

This study described PA engagement levels among adults residing in 11 countries during the COVID-19 lockdown and examined the associations of country-level COVID-19 risk, mental health symptoms, and demographic factors with PA. The main findings show that more than 40% of participants were insufficiently active and/or reported decreased PA during lockdown relative to pre-COVID-19. We also observed significant differences in PA levels during lockdown across countries, with participants residing in countries with more stringent lockdown policies having a greater likelihood of being insufficiently active. Furthermore, higher depression or anxiety symptom scores were associated with a greater likelihood of decreased PA. Greater depression symptoms were also associated with an increased likelihood of being insufficiently active during lockdown. Our findings add to existing literature on the potential impact of COVID-19 lockdown on reduced PA [18,19,20,21,22,23,24], suggesting that PA levels during lockdown may have been influenced by the stringency of governmental policy responses as well as participants’ mental health symptoms, although the directionality of these associations cannot be determined in our cross-sectional study. Given the health risks associated with physical inactivity, our findings have important implications for the development and implementation of PA promotion programs during public health emergencies when lockdown is initiated [25,26,38,39].

Although the WHO encouraged engagement in PA during lockdown [11,13,14], various barriers to do so existed, including concerns of contracting the virus in outdoor and indoor environments [4,5] the timing, length, and stringency of governmental restrictions [6,9,39,40,41,42], and confusing or inconsistent public health messages [43]. Our results revealed that the proportion of insufficient PA during lockdown in the 11 participating countries ranged from 21.1% in Bulgaria to 58.2% in Brazil. Seven of the 11 countries in our study showed a higher prevalence of insufficient PA during the COVID-19 than that of the 2016 worldwide trends in PA levels reported by Guthold et al. [44]. Additionally, we also found that participants from countries with more stringent governmental responses had greater proportions of physical inactivity during lockdown. Specifically, more than half of participants residing in Brazil, Malaysia, China, and India were insufficiently active during lockdown, with three out of these four countries having the highest average governmental policy response score among the 11 countries included in this study. These results, in line with others [18,19,20,21,22,23], suggest that COVID-19 lockdown and associated restrictions substantially reduced people’s opportunities to engage in PA by limiting access to usual places to be physically active, such as fitness and health centers, gyms, and outdoor recreation facilities [18,19,20,21,24]. However, our findings are not in agreement with some other studies that found increased PA levels during lockdown [45,46]. WHO has recommended 150 min of moderate-intensity or 75 min of vigorous-intensity PA per week, or a combination of both during lockdown. Given the rise in mental health symptoms during lockdown and significant mental and physical health benefits of PA [12,13,14,15,16], maintaining these recommended levels of PA during this period is even more important than usual [11,47].

Consistent with prior studies [18,19,20,21,22,23], we found that 45% of participants reported a decreased PA level during COVID-19 lockdown relative to pre-COVID-19. Interestingly, our findings revealed that participants engaging in low PA level during lockdown were more likely to report reductions in PA level from pre-COVID to lockdown. However, Meyer et al. [19] found that US adults reporting being active before the pandemic experienced significant PA level reductions during the pandemic. Castañeda-Babarro et al. [18] found that individuals engaging in the most vigorous PA pre-COVID showed the greatest reductions in vigorous activity time during lockdown. Although our study did not collect pre-COVID PA data, our results could have been due to similar trends. A possibility is that some of our participants who had engaged in high PA levels pre-COVID may have decreased their PA and engaged in a low PA level during lockdown as the result of the closure of recreational facilities or the lack of access to adequate spaces at home [25,26,40,47]. Another possibility is that people who were already highly intrinsically motivated to be active continued to do so creatively during the lockdown (e.g., online fitness classes, home-based physical activities) while individuals with low motivation were more influenced by extrinsic limitations [11,26,48]. Future PA promotion programs should include strategies to increase intrinsic motivation and autonomy to engage in PA during difficult times [26,48].

The relationship between mental health and PA is bidirectional [49]. While anxiety and depression may lead to decreased PA, regular PA improves mental health by reducing anxiety and depression [14,15,16]. Most existing studies examining the impacts of COVID-19 public health measures have focused primarily on the effects of PA on mental health symptoms [17,19,24,25,47] with few studies investigating the influence of mental health symptoms on PA engagement. While our cross-sectional study cannot determine the direction of the relationship, we found that higher depression and anxiety scores during lockdown were associated with decreased PA engagement. These findings support the findings of other studies demonstrating an inverse association between mental health symptoms and pandemic PA behaviors [19,21,22,23,24,25,50]. PA promotion programs should consider screening individuals for mental health problems and tailor a PA program accordingly [26,40]. Conversely, engaging in PA promotes physical and mental health and supports social connectedness and stress management, which may help individuals cope with difficulties experienced during stressful situations such as the COVID-19 pandemic [11,12,13]. Our results show more than 70% of participants reported using PA to cope with the COVID-19 lockdown. These findings, in line with WHO PA recommendations, highlight the importance of developing country-specific health communication and social marketing campaigns that promote the mental health benefits of PA [51,52] and utility of PA as a coping strategy to mitigate the psychological burden and negative emotions associated with the disease outbreak and lockdown measures [18,47,50].

Among participants in this study who reported engaging in PA during lockdown, more than 60% engaged in PA at home, nearly 40% doing so exclusively indoors, and more than 60% did so by themselves. Many participants report using online PA programs. These results are similar to the results of other studies showing that during COVID-19 lockdown, people engaged in easily practiced, home-based activities that did not require large spaces or equipment, such as aerobic exercise using stationary bikes, dance-based exercise (e.g., Zumba dancing, audio–visual-directed gymnastics), bodyweight strength training (e.g., push-ups, sit-ups), or yoga [11,13,26]. Many alternatives to traditional PA activities were also developed and implemented during the COVID-19 pandemic, including online programs that aimed to help people remain active while avoiding the risk of COVID-19 infection [13,14,38]. These online PA programs may increase PA accessibility and therefore should be promoted after the COVID-19 pandemic [21,40,53]. Such online PA programs should provide the groundwork for future PA initiatives and be disseminated via the internet and social media platforms [38]. In this regard, there is a need to develop online programs that are consistent with the United Nations Sustainable Development Goal 3 (Health and Well-Being) and Goal 11 (Sustainable Cities and Communities) [54,55]. In the last decade, the “smart city” concept has evolved and is conceptualized as having dimensions of smart technology, smart health, and smart institutions all working together to improve well-being to maintain quality of life [56,57,58]. In the future, and in times of global health crisis, smart cities that utilize technology with online PA programs must be in place in order to maintain health and well-being. The results of this study do support the need for country-specific PA interventions that are home-based and/or online to promote PA during global health emergencies.

### Limitations

There are several strengths of the present study, including the inclusion of 11 participating countries, a large sample size, a well-designed and implemented study protocol and measures, and examination of country-level COVID-19 factors for PA engagement. However, this study has several limitations that warrant attention. The results of this study should be interpreted with caution given the identified limitations of this study. First, we collected data retrospectively using self-reported measures, therefore, our findings on participants’ mental health status and PA levels were subject to recall and social desirability bias. However, it is important to recognize that the COVID-19 lockdown is an unprecedented event; people often remember their experiences, including how they felt and behaviors they engaged in, during significant life events such as the COVID-19 lockdown. Second, our data were cross-sectional so causality cannot be inferred. Third, our data were not representative of the base population in each of the 11 countries due to the use of convenience sampling; our sample included more females and younger age groups than the base population from the 11 counties. This study was also limited by the timing of data collection, with each country being in a different stage of lockdown during data collection. Thus, the results of this study may not be generalizable to other populations within or across countries; results should be interpreted within the context of these identified limitations. Fourth, we used a conservative approach for individuals with unit nonresponses to the PA questions by excluding them from analyses; thus, our results may have underestimated the number of individuals who were insufficiently active during lockdown. Finally, future prospective, longitudinal research is needed to corroborate our findings regarding changes in PA over time and to assess the impact of changes in social restrictions on PA levels.

## 5. Conclusions

The COVID-19 pandemic lockdown had a negative impact on population PA behaviors, leading to significant reductions in PA. >Our results indicate that more than 40% of participants were not sufficiently active and/or reported decreased PA during lockdown. Participants from countries with more stringent governmental policies were more likely to be insufficiently active compared to participants from countries with less stringent governmental responses. These results suggest that governmental policy responses to COVID-19 posed barriers to continuing PA. These findings emphasize the need for country-specific PA promotion interventions and suggest that governments with more stringent restrictions need to consider additional supports to promote PA engagement among their residents. These interventions should utilize creative approaches (e.g., home-based exercise, online programs) and evidence-based strategies to increase PA engagement. Public health campaigns are needed to promote PA during this and other similar global emergencies. Future research is needed on this topic, including population-based, multicountry longitudinal studies to corroborate our study findings.

## Figures and Tables

**Table 1:** Demographic characteristics and mental health symptoms of participants from the 11 participating countries (n = 11,775).

Characteristic		N (%)
Country	Brazil	1432 (12.2)
	Bulgaria	933 (7.9)
	China	1952 (16.6)
	India	848 (7.2)
	Ireland	930 (7.9)
	Malaysia	766 (6.5)
	North Macedonia	804 (6.8)
	Singapore	800 (6.8)
	Spain	962 (8.2)
	Turkey	1360 (11.6)
	United States	988 (8.4)
Sex	Female	7498 (63.7)
	Male	4170 (35.4)
	Other	107 (0.9)
Age group	18–24	3560 (30.3)
	25–34	2540 (21.6)
	35–44	2369 (20.1)
	45–54	1703 (14.5)
	55–64	1111 (9.4)
	65 years or older	480 (4.1)
Marital status	Married	5179 (44.0)
	Single	5783 (49.1)
	Other	813 (6.9)
Education level	High school degree or lower	2679 (22.8)
	Associate degree	1681 (14.3)
	Bachelor’s degree	4281 (36.4)
	Graduate degree	3111 (26.5)
General health before COVID-19	Excellent	2927 (24.9)
	Very Good	5083 (43.2)
	Good	3144 (26.7)
	Fair	621 (5.3)
Living with person(s) ≥65 years old during lockdown	Yes	2468 (21.0)
	No	9307 (79.0)
Having a child in the household during lockdown	Yes	3720 (31.6)
	No	8055 (68.4)
Required to go out of the home to work during lockdown	Full-time	2091 (17.8)
	Part-time	2202 (18.7)
	No	7482 (63.5)
Mental health symptoms during lockdown (mean, standard deviation)	Anxiety score	56.4 (9.7)
	Depression score	52.2 (9.7)

**Table 2:** Country-level COVID-19 risk factors and physical activity undertaken during lockdown, by 11 participating countries (n = 11,775).

	Insufficiently Active		Level of Physical Activity				Change in Physical Activity			Increase in Confirmed Cases ^a^	Governmental Policy Response ^a^
	Yes	No	None	Low	Moderate	High	Increase	No Change	Decrease		
	n (%)	n (%)	n (%)	n (%)	n (%)	n (%)	n (%)	n (%)	n (%)	Per million population	Standardized score
Brazil	834 (58.2)	598 (41.8)	481 (33.6)	353 (24.7)	270 (18.9)	328 (22.9)	239 (16.7)	343 (24.0)	800 (55.9)	106.3	75.6
Bulgaria	197 (21.1)	736 (78.9)	83 (8.9)	114 (12.2)	290 (31.1)	446 (47.8)	75 (8.0)	448 (48.0)	400 (42.9)	14.0	51.7
China	1064 (54.5)	888 (45.5)	618 (31.7)	446 (22.9)	405 (20.8)	483 (24.7)	329 (16.9)	844 (43.2)	779 (39.9)	0.3	73.3
India	435 (51.3)	413 (48.7)	362 (42.7)	73 (8.6)	178 (21.0)	235 (27.7)	172 (20.3)	265 (31.3)	348 (41.0)	15.7	82.9
Ireland	233 (25.1)	697 (75.0)	92 (9.9)	141 (15.2)	274 (29.5	423 (45.5)	336 (36.1)	238 (25.6)	314 (33.8)	34.2	67.6
Malaysia	423 (55.2)	343 (44.8)	265 (34.6)	158 (20.6)	123 (16.1)	220 (28.7)	129 (16.8)	258 (33.7)	379 (49.5)	1.6	61.6
North Macedonia	240 (29.9)	564 (70.2)	99 (12.3)	141 (17.5)	163 (20.3)	401 (49.9)	152 (18.9)	326 (40.6)	324 (40.3)	40.8	65.5
Singapore	342 (42.8)	458 (57.3)	140 (17.5)	202 (25.3)	188 (23.5)	270 (33.8)	241 (30.1)	352 (44.0)	207 (25.9)	52.9	60.5
Spain	411 (42.7)	551 (57.3)	213 (22.1)	198 (20.6)	252 (26.2)	299 (31.1)	198 (20.6)	202 (21.0)	524 (54.5)	54.4	65.3
Turkey	604 (44.4)	756 (55.6)	289 (21.3)	315 (23.2)	342 (25.2)	414 (30.4)	206 (15.2)	350 (25.7)	804 (59.1)	19.1	64.2
United States	386 (39.1)	602 (60.9)	138 (14.0)	248 (25.1)	307 (31.1)	295 (29.9)	222 (22.5)	328 (33.2)	394 (39.9)	100.1	66.8
Overall	5169 (43.9) ^†^	6606 (56.1)	2780 (23.6)	2389 (20.3)	2792 (23.7)	3814 (32.4) ^†^	2522 (21.4)	3175 (27.0)	5273 (44.8) ^†^	39.4	67.7 ^§^

^†^ Chi-square tests were conducted for the associations between country and insufficiently active, (X^2^ = 270.8, *p* < 0.0001), level of physical activity (X^2^ = 356.21, *p* < 0.0001), and change in physical activity (X^2^ = 675.02, *p* < 0.0001). **^§^**Pearson correlation coefficient was calculated between insufficiently active and an increase in confirmed cases (*r* = −0.017, *p* = 0.065), and governmental policy responses (*r* = −0.157, *p* < 0.0001). ^a^ Data were based on Our World in Data. [34]

**Table 3:** Factors associated with physical activity during lockdown.

Factors	Insufficiently Active		Decrease in Physical Activity
	OR (95% CI)		OR (95% CI)
Country-level COVID-19 risk	10-unit change of confirmed cases	0.97 (0.90, 1.05)	0.98 (0.92, 1.04)
	5-unit change of policy response score	1.22 (1.03, 1.45)	1.01 (0.88, 1.15)
Mental health symptoms during lockdown	10-unit change of anxiety score	0.98 (0.92, 1.05)	1.18 (1.11, 1.26)
	10-unit change of depression score	1.44 (1.07, 1.22)	1.15 (1.08, 1.22)
Sex ^a^	Female	1.30 (1.19, 1.42)	0.92 (0.85, 1.00)
	Male	Reference	Reference
Age group	18–24	Reference	Reference
	25–34	1.15 (1.01, 1.30)	1.06 (0.93, 1.20)
	35–44	1.16 (0.99, 1.36)	1.19 (1.03, 1.39)
	45–54	1.09 (0.92, 1.29)	1.24 (1.05, 1.46)
	55–64	0.98 (0.81, 1.18)	1.37 (1.14, 1.64)
	65 years or older	1.50 (1.18, 1.91)	1.10 (0.87, 1.40)
Marital status ^a^	Married	1.15 (1.02, 1.31)	0.98 (0.87, 1.11)
	Single	Reference	Reference
Education level	High school degree or lower	Reference	Reference
	Associate degree	1.01 (0.88, 1.15)	1.06 (0.93, 1.21)
	Bachelor’s degree	0.79 (0.71, 0.88)	1.20(1.08, 1.33)
	Graduate degree	0.79 (0.70, 0.89)	1.30(1.16, 1.47)
General health before COVID-19	Excellent	0.34 (0.28, 0.41)	1.00 (0.83, 1.20)
	Very Good	0.47 (0.39, 0.56)	1.18 (0.99, 1.40)
	Good	0.72 (0.59, 0.86)	1.17 (0.97, 1.40)
	Fair	Reference	Reference
Living with person(s) ≥65 years old during lockdown	Yes	0.96 (0.86, 1.06)	0.93 (0.84, 1.03)
	No	Reference	Reference
Having a child in the household during lockdown	Yes	1.17 (1.05, 1.31)	0.89 (0.80, 0.99)
	No	Reference	Reference
Required to go out of the home to work during lockdown	Full-time	1.16 (1.04, 1.30)	0.91 (0.81, 1.01)
	Part-time	1.03 (0.93, 1.14)	1.00 (0.90, 1.11)
	No	Reference	Reference
ICC (null model) ^b^		0.075	0.045
AIC (full model) ^b^		14,798.77	15,369.79
BIC (full model) ^b^		14,808.72	15,379.73

Note: Results were based on two separate hierarchical generalized linear models with a dichotomous outcome variable that assessed the factors associated with the outcomes of (1) insufficiently active, and (2) a decrease in physical activity during lockdown. Abbreviations: ICC: intralevel correlation coefficient; AIC: Akaike information criterion; BIC: Bayesian information criterion; CI: confidence intervals. ^a^ Estimates for participants that reported “Other” on variables of sex and marital status were not listed. ^b^ Results from null model tests were not listed, except for the ICC from null models. Null models were conducted without fixed factors but a random intercept of the country variable. For insufficiently active outcome, null model n = 11,746, ICC Country = 0.075, AIC = 15,473.24, and BIC = 15,474.04; for decrease in PA engagement outcome, null model n = 11,746, ICC Country = 0.045, AIC = 15,773.85, and BIC = 15,774.65.

## Data Availability

Anonymized data used and/or analyzed during the current study, along with detailed study protocol, are available from the corresponding authors on reasonable request. The data are not publicly available due to privacy restrictions.

## Global Community Health-COVID-19 Collaborative Research Team

Brazil (Carolin Lusby, Roberto V. Mossa, Larissa C. Pires, William F. Santana, Ricardo R. Uvinha), Bulgaria (Daniela S. Dasheva, Alixander J. Rocha), China (Yuwei Cai, Jiaai Huang, Rashid Menhas, Xuehui Sang, Yu Weng), India (Komal Chandiramani, Nudrat Jahan, Meena Osmany, Sonia), Ireland (Ursula Kenny), North Macedonia (Mishko Djidrov, Gordana Miteva, Katerina Mitevska Petrusheva, Snezana Jovanova-Mitkovska), Malaysia (Nur Syamsina Ahmad, Mohd Rahimi Che Jusoh, Yee Cheng Kueh, Ayu Suzailiana Muhamad, Marilyn Li Yin Ong), Singapore (Peggy Boey, Taufiq I. Jamaludin, Yew Cheo Ng, Edward J. Say, Chea Nuan Yow), Spain (Carina Soledad González-González, Myriam Guerra-Balic, Sarah Muñoz-Violant, Manuel J Rodriguez-Allue, Oriol Sansano-Nadal), Turkey (Bijen Filiz, Bengu Guven, Ezel Nur Korur, Funda Coskun Ozyol, Yilmaz Yuksel), and United States (Laura M. Dattner, Amanda K. Hautmann)
